# What competencies do European general practice trainees value the most? A prioritisation exercise using a Delphi-informed approach

**DOI:** 10.1080/14739879.2023.2222718

**Published:** 2023-07-16

**Authors:** Helene Junge, Aaron Poppleton, Sophie Sun, Szidonia Janos, Fabian Dupont

**Affiliations:** a Department of Family Medicine, Saarland University, Homburg, Germany; b School of Medicine, Keele University, Keele, UK; c Collège Universitaire de Médecine Générale, Université Claude Bernard, Lyon, France; d Department of Family Medicine, University of Medicine and Pharmacy Iuliu Haţieganu Cluj-Napoca/klausenburg, Romania

**Keywords:** general practice, competencies, speciality training, curriculum, medical education

## Abstract

General Practice has changed over the past decade. Expansion of clinicians’ roles may create uncertainty, stress, and overload – particular for those at the start of their career. The WONCA Europe network for medical education, EURACT, has published competency-based aims and requirements for speciality training in general practice. Greater understanding of the trainee perspective would support planning and delivery of postgraduate training curricula. This two-step study aims to provide a competency priority list, created by European early career general practitioners, to highlight skills that this generation considers highly essential in future speciality training. A competency list was drafted with trainee- and early career general practitioners from across Europe at the Vasco da Gama Movement Forum (Edinburgh, January 2022). Participants identified competencies that they regarded as most relevant for future speciality training in their respective national contexts. Competencies were coded into categories and ranked in two consecutive rounds, the first taking place online and the second at WONCA Europe (London, June 2022). After two rounds, a consensual list of three main competencies for each category was drafted. The top three competencies for each category remained the same throughout both rounds and may be considered competencies that early career general practitioners in Europe consider important for training. Prioritisation of these competencies by institutions and educators within general practice training programmes may support trainees’ satisfaction and perceived preparedness for practice.

## Introduction

General practice (GP) has changed dramatically over the past few decades. The COVID−19 pandemic has further accelerated changes in workload for general practitioners (GPs), affecting both patient care and postgraduate speciality training. In response to these diverse, evolving challenges, competency-based medical education (CBME) has been of growing importance, particularly for GP [1,2]. CBME creates intended learning outcomes (ILOs) based on real-world skills within the medical workplace. Practitioner skill proficiency is intended on completion of a competency-based curriculum [2]. To date, postgraduate GP curriculum design and creation of ILOs have primarily been tasks carried out by institutions and educators. In 2018, Michels et al. summarised core competencies that should serve as overarching ILO in GP training according to the World Organization of Doctors of Family Medicine (WONCA) and the European Academy of Teachers of General and Family Medicine (EURACT) [3,4]. According to Michels et al., there are three main actors in GP speciality training: a) trainees, b) trainers and c) training institutions. The trainers, in this case represented by WONCA and EURACT, highlight six core competencies, namely (1) Primary Care Management, (2) Person-Centred Care, (3) Specific Problem-Solving Skills, (4) Comprehensive Approach, (5) Community Orientation, and (6) Holistic Approach. These core competencies are further subdivided into Twelve ‘central characteristics of the discipline of GP’ [4]. Together with the three ‘additional characteristics of GP’ (science, attitude and context) they are summarised in the widely cited WONCA tree [4,5]. A needs-analysis conducted in 2012 among both novice and expert GP educators found that educators coming from different European countries and different health care systems reported several similar problems, needs and expectations in regard to their role as GP educators [6]. In a 2017 study, EURACT representatives stated that establishing common ILOs for speciality training is necessary to strengthen European GP [7]. Greater inclusion of trainee and early-career GPs in the development of the GP speciality training curriculum and teaching process has been proposed yet remains uncommon in practice [4]. GP trainees’ view on which competencies should be focused on in future GP speciality training has not been investigated, especially not at a European level. This study provides a pan-European structured priority list of important competencies for future GP practice from the perspective of young GPs and GPs in training.

## Methods

### Design and participants

The study follows a two-step systematic mixed methods approach inspired by the Delphi technique. The Delphi technique is a structured multi-stage survey and a widely accepted qualitative method in medical education research, especially in situations when evidence is lacking [8,9]. Through consecutive rounds of questioning, it aims to build consensus among a group of experts. While this study cannot formally be considered a Delphi study, since it did not interview the same group of experts in each round, it was informed by the Delphi method. Delphi studies usually begin by distributing an open-ended questionnaire to experts on a particular topic. Items or suggestions listed by experts are translated into general statements, with duplicates removed. Experts then vote on the importance of the individual items in subsequent rounds [10,11]. In this study, the Delphi approach was modified by replacing the open-ended questionnaire with an inductive Town Hall discussion. Another in-person discussion was held before the second survey round. These in-person discussions enable experts to clarify the points raised, avoid misunderstandings, and may increase response rates in subsequent rounds [12]. Anonymity was maintained throughout the voting process [12,13]. The study participants were early-career doctors in GP as defined by the Vasco da Gama Movement (VDGM) (GPs during and within Five years of completion of GP specialist training). Participants were recruited through participation in voluntary workshops at two GP-focused conferences (VdGM Forum 2022, VdGM pre-conference for WONCA Europe 2022) or contacted as VdGM council members.

### Ethical considerations

Ethical approval was obtained prior to study initiation (Saarland medical association ethics committee; 25.09.2020 (Bu234/20)). All participants were informed of the study design before participation. Written consent to participation, recording of verbal contributions, and anonymous data collection was obtained digitally through Sli.do™ and Qualtrics ™ (free version), respectively, at the beginning of each survey round or face-to-face discussion. Submission of email address was required to participate in consecutive rounds. Providing this information was voluntary, with participants informed that they waived anonymity by doing so.

### Data collection strategy

The first stage of data collection took place at the 7th Vasco da Gama movement (VdGM) Forum in Edinburgh on Date January 28, 2022. Participants were informed about the study structure and Bloom’s taxonomy as a tool to categorise competencies [14]. A hybrid Town Hall meeting was then held using the digital word-cloud tool Sli.do™. Via Sli.do™, suggestions were recorded digitally and fed back to the participants in real time with the help of a PowerPoint presentation. The in-person discussion was structured by the moderators (HJ, FD) based on Bloom’s taxonomy domains to ensure all areas of competencies were covered in the discussion and received the same attention throughout the workshop. Experts’ suggestions on the most important competencies (psychomotor, cognitive, and affective) were collected digitally via Sli.do™. Experts were invited to suggest competencies they considered to be essential for current and future GP practice. No suggestions on competencies were made by the moderators to encourage open brainstorming by the experts. Simultaneous feedback and follow-up questions were asked to the audience to stimulate further explanations of contributions until theoretic saturation was reached. Participants could then register their email to participate in the decentralised first survey round. Sli.do™ poll results were extracted, coded, and grouped through authors discussion (HJ, AP and FD). The verbal discussion was re-evaluated multiple times before and after coding to ensure keywords were assigned to the correct group. No new items were added during the analysis. Participant wording was retained wherever possible. All competencies seemingly assigned to the incorrect domain by participants were discussed by study authors and reassigned based on mutual team consensus. Six competency subgroups were conceived for the ‘psychomotor’ and ‘affective’ domains, with seven subgroups for the ‘cognitive’ domain due to the large number and variety of contributions. Subgroups were listed in a ranking poll using an online survey tool (Qualtrics™).

### First survey round

All individuals providing contact details during the VdGM workshop (28/01/2022) (*n* = 13), VdGM national representatives and executive council members (*n* = 46) were invited to participate in the first survey round via e-mail or WhatsApp™. Participation constituted of completion of a Qualtrics™ online survey over a four-week period (11/02/2022–07/03/2022). Participants were instructed to rank an unsorted list of GP training competencies, which consisted of experts’ contributions from the Town Hall Meeting. Ranking was based on their perceived importance of the competencies for current and future GP training (scale 1–6 or 1–7; 1 = greatest importance, 6/7 = least importance) to their perceived importance within GP speciality training (scale 1–6 or 1–7; 1 = greatest importance, 6/7 = least importance). Participants could suggest additional competencies or modifications to those provided. A single reminder via email was sent to all participants. Survey responses were only analysed if consent was provided and if the ranking was completed for at least one of the three categories of competencies.

### Second survey round

The second survey round took place during a WONCA Europe pre-conference workshop (London; 27/06/2022). An email reminder to study participants was sent (20/06/2022) to encourage workshop attendance. Workshop content included a short reminder of study structure, preliminary results from the first survey round, and facilitated group discussion enabling member checking. Remaining open questions from experts concerned the definitions of certain competencies. These were answered sufficiently by the authors (HJ, FD and AP). Real-time online ranking was performed using Sli.do™. Experts ranked the listed competencies according to their perceived importance for current and future GP training (scale 1–6 respective 1–7; 1 = greatest importance, 6/7 = least importance).

### Data analysis

Quantitative analyses were performed using Jamovi (Version 1.6.23.0). Descriptive statistics included mean and standard deviation (μ ± SD). Shapiro-Wilk test was used to test for normality. The level of agreement (LoA) as described by De Loe et al. was calculated to quantify the degree of consensus among participants using an approach adapted for a 7-point scale [15,16]. High LoA was assumed if 70% of the scores were given in 2 (of 7) contiguous scale levels or if 80% of the scores are given in 3 (of 7) contiguous scale levels. Skewness was calculated to test for unidirectionality of experts’ voting.

## Results

### Demographics

Thirty experts attended the first Town Hall discussion (28/01/2022). Twenty-three (77%) actively participated in the digital data collection process. Participants provided consent via Sli.do™ (*n* = 22) or email (*n* = 1) for data collection. All twenty-three participants worked in GP, six (26%) were GP trainees, eleven (48%) were within Five years and six (26%) more than five years after speciality training.

VdGM council members and all Town Hall meeting participants providing their contact details were invited to participate in the first digital survey round. Twenty-nine completed the survey and were included in data analysis. Participants were from Twenty-two different countries (see figure). Most of the participants came from France (*n* = 3, 10.3%), United Kingdom (UK) (*n* = 3, 10.3%) and Spain (*n* = 3, 10.3%). Most participants had completed GP speciality training (*n* = 18, 64%). One participant did not provide her/his training status.

**Figure 1.**
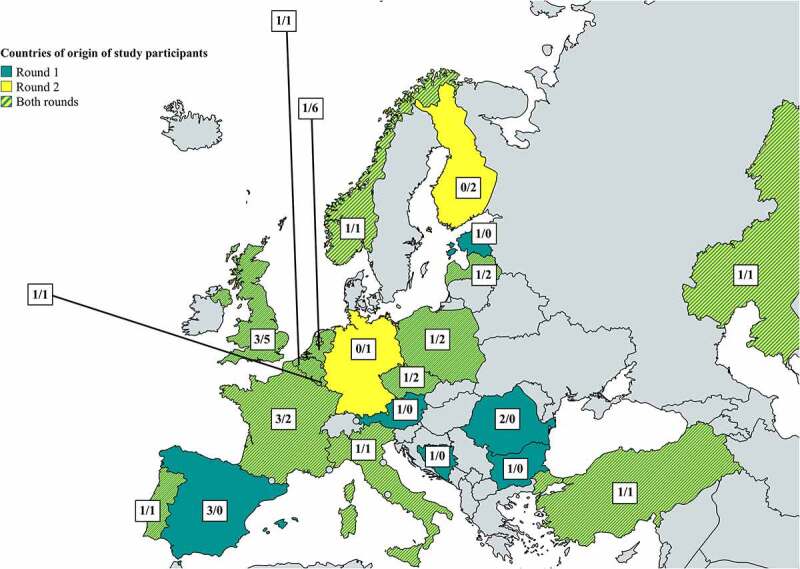
Countries of origin for participants in the survey rounds and number of participants per round (Round 1/Round 2). not on map: Kyrgyzstan (1/0), Malta (1/0), Israel (1/1) [24].

Thirty-six individuals attended the workshop for the second survey round (30/06/2022). Thirty-three (92%) participated in the digital data collection process. All participants provided consent via Sli.do. The participants were from seventeen countries (see Figure 1), the most common being the Netherlands (*n* = 6, 18%) and UK (*n* = 5, 15%). Most were in GP speciality training (*n* = 19, 58%).

### First survey round

#### Psychomotor competencies

Twenty-eight participants ranked the psychomotor competencies. ‘General physical examination skills’ had the lowest average competency score (1.32, SD: 0.945) and the greatest skewness (3.25), which indicates participants consider this competency to be highly relevant for GP training. This was followed by ‘specific examination skills’ and ‘documentation and digital skills’ (see Table 1). Overall, skewness was positive for competencies ranked with high importance and negative for competencies ranked with low importance, indicating unidirectionality. All rankings for psychomotor competencies had high or medium LoA.

**Table 1.** Psychomotor competencies (PC), cognitive competencies (CC), affective competencies (AC) and their rank, level of agreement (LoA) and skewness in round 1 of the study. Green colour: high or medium LoA. Yellow colour: low LoA. Red colour: no consensus.

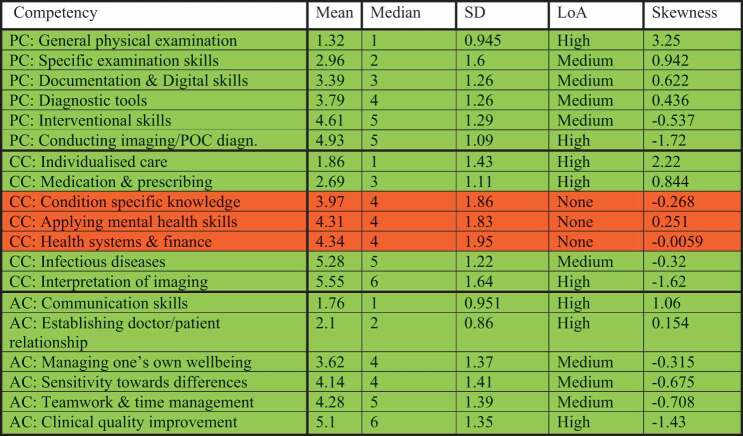

#### Cognitive competencies

Twenty-nine participants ranked for cognitive competencies. ‘Individualised care’ was ranked as the most important cognitive competency, followed by ‘medication and prescribing’ and ‘condition specific knowledge’. A high or medium LoA was found for 57% of the competencies. No agreement was found for ‘health system and finance’, ‘condition-specific knowledge’ and ‘mental health skills’.

#### Affective competencies

Twenty-nine participants ranked affective competencies. ‘Communication skills’ were ranked as the most important cognitive competency, followed by ‘doctor/patient relationship’. Despite a significant discussion of ‘managing personal wellbeing’ during the workshop in Edinburgh, its average rank of 3.62 suggests a moderate importance (see Table 1). A high or medium LoA was found for 84% of the affective competencies.

### Second survey round

#### Psychomotor competencies

Thirty-one participants ranked psychomotor competencies. The top two competencies remained unchanged between the first and second survey round (see Table 2). ‘Documentation and digital skills’ fell from third to fifth rank. Competency order remained otherwise unchanged. Skewness was positive for competencies ranked with high importance and low for competencies ranked with low importance, indicating unidirectionality. All rankings for psychomotor competencies had high or medium LoA.

**Table 2.** Psychomotor competencies (PC), cognitive competencies (CC), affective competencies (AC) and their rank, level of agreement (LoA) and skewness in round 2 of the study. Green colour: High or medium LoA. Yellow colour: low LoA. Red colour: no consensus.

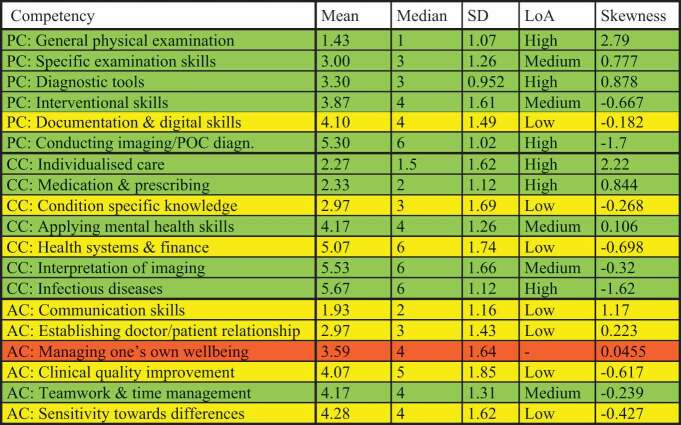

#### Cognitive competencies

Thirty-one participants ranked cognitive competencies. Results from the first survey round were mostly confirmed: the first five ranks remained unchanged. ‘Infectious diseases’ was now considered the least important of the seven cognitive competencies. 71% of the cognitive competencies had high or medium LoA.

#### Affective competencies

Thirty-one participants ranked affective competencies. The top competencies remained unchanged (‘communication skills’, ‘doctor/patient relationship’ and ‘managing personal wellbeing’). ‘Clinical quality improvement’ increased to rank four. The remaining two competencies had the same mean rank (4.16, see Table 2). Most affective competencies had a low LoA, with the exception of ‘teamwork and time management’ (medium LoA) and ‘managing one’s own wellbeing’ (no agreement).

## Discussion

This is the first study in which GP trainees from across Europe have ranked GP training competencies based on perceived importance. This list of competencies could help GP educators identify areas of speciality training requiring greater focus and adaptation to GP trainees. Individual competencies require the ability to combine skills, knowledge and attitude in a way that is helpful to dealing with a situation [17]. As such, we acknowledge an overlap between the domains of Bloom’s taxonomy and the artificial perspective it offers on workplace activities. A key element of planning a curriculum is to allocate competencies to ILOs and constructively align them in a curricular blueprint [17,18]. This is an important consideration when evaluating the compiled list.

When discussing psychomotor competencies, it is striking that high LoA was reached in both survey rounds. There seems to be a clear consensus among young GPs that general physical examination is the most important psychomotor competency obtained in training. Young GPs also seem to agree that other examination techniques as well as interventional- and diagnostic skills are important competencies for GP. This could indicate that the image of GP is strongly influenced by the idea of a ‘hands-on examiner’ in various European countries. However, it may be necessary to consider local factors in curriculum design, such as a potentially greater importance of competencies like ‘condition specific knowledge’ or ‘imaging interpretation skills’ in rural localities [19,20].

It is remarkable that the highest ranking ‘cognitive competency’ is also the most complex [14]. ‘Individualised care’ requires critically appraisal of guidelines, analysis of patient needs, activation of factual knowledge, evaluation of treatment options and their appropriateness in the current circumstance. This implies that young GPs are more concerned with the application of knowledge to an individual patient than accumulating detailed disease-focused knowledge itself. We feel this is an important issue worthy of increased attention in GP speciality training. Interestingly, the experts in this study ranked ‘health systems and finance’ in the middle of the competencies in both rounds, with low LoA. This may suggest that GP trainees see themselves as more than medical specialists, e.g. as mediators between patients and the health system. It may also indicate that the roles, responsibilities, and associated training need for this competency vary significantly across Europe.

Affective competencies stimulated the greatest discussion and showed the lowest LoA of the competency categories. This could be due to the range of countries represented and associated variations in medical education. Furthermore, personal factors, cultural background and the ‘hidden curriculum’ may have influenced views on ‘managing one’s own wellbeing’ and ‘sensitivity towards differences’ [21]. Affective competencies have traditionally received comparatively little attention in medical education [22]. Young GPs may have received little formal training in this area. Our oral discussions suggest great interest in this area. Future research is needed to identify the preferences and requirements of young GPs regarding the teaching of affective skills and its value in GP training.

Our results show overlap with previous WONCA and EURACT recommendations regarding GP speciality training. The WONCA tree incorporated aspects of ‘communication skills’, ‘doctor–patient relationship’ and ‘individualised care’ under ‘person-centred care’. The core WONCA competency ‘holistic modelling’ is further described as the ability to consider ‘problems in their physical, psychological, social, cultural and existential dimensions’ [4]. This corresponds with the request of GP trainees that ‘sensitivity towards differences’ should be included as an affective competency in GP training. Regular reassessment of GP trainees’ perceived training needs is required for quality assurance and to detect changes over time [23].

We recognise that the list can only cover a fraction of GP competencies and risks, giving the impression that lower-ranking competencies are not important. The current study aimed to identify competencies of greatest perceived importance by young GPs, rather than repeat or rearrange existing GP training curricula.

## Limitations

We acknowledge the likely selection bias in this study. All meetings and ranking processes were in English. Two of the three survey rounds were in-person conference workshops. Attending experts will have had language skills, time, financial resources and potentially a special interest in CBME. Both conferences took place in the UK, meaning more experts were Western European. We recommend steps to include the preferences of financially disadvantaged and Eastern European young GPs in future research, e.g. digital and multilingual options.

Even though the Town Hall discussion and the two survey rounds targeted the same cohort of experts, it cannot be assumed that the same individuals took part in all rounds of the study. By describing the socioeconomic data of the experts and targeting the limited group of EYFDM-associated GPs, it was attempted to ensure similar expert groups in all survey rounds. Nevertheless, this study cannot be formally considered a Delphi study.

## Conclusion

This is the first study in which young GPs’ have prioritised aspects of GP speciality training in terms of their perceived importance. The identified priority list of psychomotor, cognitive, and affective competencies shows partial overlap with previous EURACT and WONCA guidance. Affective competencies including ‘managing one’s own wellbeing’ and ‘sensitivity towards differences’ had low levels of ranking agreement and stimulated significant discussion. Greater understanding of young GPs' preferences and attitudes towards affective competencies is required. GP educationalists should ensure that competencies, especially those relevant to young GPs, are included and focussed on in the future GP curricula.

## Abbreviations

CBME: Competency-based medical education
EURACT: European Academy of Teachers in General Practice
GP: general practice
GPs: general practitioners
LoA: level of agreement
WONCA: World Organization of Family Doctors
VdGM: Vasco da Gama Movement
